# Effects of the Intensity of Leg Isometric Training on the Vasculature of Trained and Untrained Limbs and Resting Blood Pressure in Middle-Aged Men

**DOI:** 10.1155/2012/964697

**Published:** 2012-09-09

**Authors:** Anthony W. Baross, Jonathan D. Wiles, Ian L. Swaine

**Affiliations:** ^1^Sport and Exercise Science, University of Northampton, Park Campus, Boughton Green Road, Northampton NN2 7AL, UK; ^2^Sport and Exercise Science, Canterbury Christ Church University, Canterbury, Kent CT1 1QU, UK

## Abstract

The purpose of this study was to establish whether changes in resting blood pressure and the vasculature of trained and untrained limbs are dependent on training intensity, following isometric-leg training. Thirty middle-aged males undertook an 8 week training programme (4 × 2 min bilateral-leg isometric contractions 3 times per week). Two groups trained at either high (HI; 14%MVC) or low (LO; 8%MVC) intensity a third group (CON) acted as controls. All parameters were measured at baseline, 4-weeks and post-training. Resting SBP (−10.8 ± 7.9 mmHg), MAP (−4.7 ± 6.8 mmHg) and HR (−4.8 ± 5.9 b*·*min^−1^) fell significantly in the HI group post-training with concomitant significant increases in resting femoral mean artery diameter (FMAD; 1.0 ± 0.4 mm), femoral mean blood velocity (FMBV; 0.68 ± 0.83 cm*·*s^−1^), resting femoral artery blood flow (FABF; 82.06 ± 31.92 ml*·*min^−1^) and resting femoral vascular conductance (FVC, 45%). No significant changes occurred in any brachial artery measure nor in any parameters measured in the LO or CON groups. These findings show that training-induced reductions in resting blood pressure after isometric-leg training in healthy middle-aged men are associated with concomitant adaptations in the local vasculature, that appear to be dependent on training intensity and take place in the later stages of training.

## 1. Introduction

Previous studies have consistently shown reductions in resting blood pressure following isometric training [1–4]. However, it is not fully understood whether these reductions are accompanied by changes to the local vasculature or what role exercise intensity has in adaptations to this type of training. Most studies have focussed on the changes in resting blood pressure after training at a given intensity, mostly involving arm training at 30% maximum voluntary contraction (MVC) [1, 2, 5, 6] rather than examining the role of exercise intensity in the adaptations to training. Two studies have investigated the effects of isometric training intensity on reductions in resting blood pressure, one using handgrip [1] and the other using double-leg extension [4]. However, it is difficult to interpret the role of training intensity in the study by Wiley et al. [1] because they reported two different training studies, performed at intensities of 30% and 50% MVC, with differing lengths of contractions (4 × 2 min, 4 × 45 s) and recovery times (3 min, 1 min) for periods of 8 and 5 weeks, respectively. 

The investigation by Wiles et al. [4] is the only study to prospectively investigate the effects of different isometric training intensities on the training-induced reductions in resting blood pressure. It appears from the data presented in their study (Figure 3), that most of the reductions in resting blood pressure occurred in the first 4 weeks in their higher-intensity training group (approximately 20% MVC), whereas the changes in the lower-intensity group (approximately 10% MVC) took 8 weeks. Indeed, in a subsequent study by the same group [7], higher-intensity double-leg training produced significant reductions in resting blood pressure after 4 weeks. Interestingly, blood pressure reductions associated with aerobic training (dynamic) are not thought to be dependent on exercise intensity [8, 9]. Taken together, these studies suggest that isometric training intensity might be important in the adaptations seen on the completion of such training, and in the rate at which they occur. 

The mechanism by which the isometric training-induced reduction in resting blood pressure is achieved remains unclear. Studies using dynamic exercise to elicit reductions in resting blood pressure have reported concurrent adaptations in both local and/or systemic vasculature [10] that seemingly lowers total peripheral resistance [11]. These changes have been associated with modified sympathetic nerve activity, increased artery lumen diameter and improved endothelium-dependent vasodilatation [10, 12]. Recently, Tinken et al. [13] also reported elevated nitric oxide (NO) levels following extended exercise training (≥8 weeks) which are thought to mediate vascular remodelling [13, 14] resulting in an increased lumen diameter [11, 15]. However, few studies to date have identified changes in resting vascular dimensions or blood flow after isometric training. Previous isometric training studies have shown alterations in central artery compliance [2, 16] and improved endothelium-dependent vasodilatation [5], but attempts have not been made to relate these changes to the intensity of training. Therefore, the aims of this study were to determine whether reductions in resting blood pressure are associated with adaptations in the muscle vasculature (of trained and untrained limbs) and whether the time course of these adaptations, is training intensity dependent. 

## 2. Materials and Methods

### 2.1. Subjects

Three groups of sedentary and nonmedicated healthy men volunteered for the study. These individuals did not engage in regular or structured exercise sessions and were classified as having a low level of physical activity following completion of the International Physical Activity Questionnaire. The participants were then randomly assigned to a training or control group. The first training group (HI) undertook isometric training at an intensity equivalent to 14% MVC (85% of their peak heart rate obtained during an incremental isometric exercise test; *n* = 10). The second group (LO) trained at an intensity that was equivalent to 8% MVC (70% of their peak HR; *n* = 10) and the final group acted as controls (CON; *n* = 10). After receiving institutional ethical approval, each participant received a detailed information sheet explaining the experimental protocol and potential risks involved then signed and completed a consent form and pretest medical questionnaire.

### 2.2. Equipment

All tests were performed in a consistent laboratory environment using an isokinetic dynamometer (Biodex Medical Systems, Inc., New York, USA). The dynamometer was fitted with an extended leg attachment to enable double-leg isometric extensions to be performed. To allow for precise synchronisation of torque, electromyography (EMG), heart rate blood pressure, and all data generated by the dynamometer were transferred to the MP150WS Biopac data acquisition system using Acknowledge (v4) software (Biopac, CA, USA) via an analogue output. All other data (EMG, heart rate, and blood pressure) were recorded using the Biopac data acquisition unit with a sampling frequency of 2000 Hz. 

For the purposes of isometric training, EMG was measured from the vastus lateralis (VL) of both legs during double-leg extension using active bipolar surface electrodes (MP-A2 Biopac, CA, USA) with a 25 mm interelectrode distance. The electrodes were placed approximately one-third of the muscle length above the patella and orientated parallel to the direction of the underlying muscle fibres [17]. The EMG signal was amplified (gain = 300, input impedance = 10 G*Ω*) by the active electrodes and directed to a high-level transducer (HLT100C Biopac, CA, USA). The EMG was further filtered using 20 Hz high-pass and 500 Hz low-pass filters, smoothed with a 250 ms moving average and was root mean squared. The root mean squared values from both limbs were then averaged to give a single root mean squared EMG value. 

Blood pressure (systolic, diastolic, and mean arterial pressure) was also continually measured using a noninvasive blood pressure (NIBP) monitor (NIBP100A, Biopac, CA, USA). The mean pressure values of the pressure waveform are displayed on a monitor via a sensor positioned percutaneously across the radial artery at the distal edge of the radius with the reading being updated approximately every 10 seconds. The use of this type of blood-pressure monitoring has been assessed for accuracy and reliability of measurement [18] and has been reported to be an acceptable tool for measuring blood pressure. Coefficients of variation, expressed as a percentage of the mean, ranged from systolic 2.2% to diastolic and 3.0% indicating a high reliability. Resting blood pressure measures were recorded for 60 seconds following 15 minutes of rest, with the participants in the supine position. Heart rate was recorded continuously through a single-lead electrocardiogram using pregelled disposable electrodes (EL501, Biopac, CA, USA). 

An ultrasound video imaging system (LOGIQ Book XP, General Electric, Bedford, UK) in the pulse wave Doppler mode, equipped with a 11 mm diameter linear transducer probe (8L-RS, General Electric) operating at an imaging frequency of 8 MHz with a variable Doppler frequency of 4–10 MHz was used to measure two-dimensional (2D) femoral and brachial artery diameter and mean arterial velocity simultaneously, at rest. All continuous mean blood velocity measures throughout the investigation were taken with a Doppler transducer probe insonation angle of 60°. When recording femoral measures, the probe was positioned over the common femoral artery distal to the inguinal ligament and above the bifurcation into the superficial and profunda femoral branch. For the brachial artery, the probe was placed over the brachial artery approximately 9 cm proximal to the medial epicondyle. The ultrasound gates were adjusted to the width of the artery to ensure complete insonation of the artery. Arterial diameter was determined by a perpendicular measure from the lumen-intima interface of the rear wall to the lumen-intima interface of the far wall of the vessel. Vascular diameter and velocity measure were determined as the mean of three measures collected at 20 s intervals during the 1 minute data collection period and analysed by the same investigator [19, 20]. The mean arterial diameter and mean blood velocity measures were processed by the ultrasound software to generate a mean blood flow value. Vascular conductance was calculated from mean blood flow and mean arterial pressure measures. The day-to-day reliability of these measures was determined prior to the start of the study. The CV for the two trials was; 2.42 (femoral) and 3.06 (brachial) mean artery diameter, 3.19 (femoral) and 3.45 (brachial) for mean blood velocity and 2.76 (femoral) and 3.73 (brachial) for mean blood flow. 

#### 2.2.1. Training

Isometric training was performed in the same way as detailed previously [4, 21]. Briefly, participants performed an MVC test and then an incremental isometric exercise test. The training was performed on 3 days per week for a period of 8 weeks, with training sessions at least 24 hours apart, using target EMG values that equated to either 85% (HI) or 70% (LO) of the peak heart rate (HR_peak_) achieved during the final exercise intensity of the incremental test. Each training session comprised 4 × 2-minute bouts interspaced with two-minute rest periods. During all training sessions EMG, heart rate, blood pressure, and torque were continually measured and recorded. The target EMG values were recalculated following a repeat of the incremental isometric exercise test at the end of week four. This allowed for adjustments to the intensity as training progressed and took account of any adaptations which may have occurred over the initial four weeks of training. 

#### 2.2.2. Resting Measures

 Following the initial familiarisation session and prior to the first incremental isometric exercise test, participants resting heart rate and blood pressure measures were recorded. Also, brachial and femoral artery blood flow, blood velocity, artery diameter, and venous compliance were measured and recorded. These measures were subsequently recorded at the end of week 4 and after-training.

### 2.3. Data Analysis

The data were assessed for normal distribution and satisfied parametric assumptions [22]. Statistical analysis was performed using Microsoftcel, SPSS 15. It has been reported [6] that the magnitude of the blood pressure reductions are related to the initial resting values. Therefore, analysis of covariance (ANCOVA) was used to determine if there was a significant difference in the mid and after-training blood pressure compared to the baseline measures, using the baseline values as the covariate. Posthoc analysis (Bonferroni) was then used to further determine specific significant differences. Microsoft GraphPad Prism software, which uses ANCOVA to compare the slopes and intercepts of the linear regression lines, was used to analyse the data from the incremental isometric exercise test. An alpha level of 0.05 was accepted as being significantly significant.

## 3. Results

### 3.1. Baseline Data

Baseline data indicated that there were no significant pre-training differences between groups (HI versus LO versus CON) for maximum voluntary contraction (MVC), body mass, resting heart rate (RHR), resting systolic (RSBP), diastolic (RDBP), mean arterial blood pressure (RMAP), femoral artery blood flow (FABF), femoral mean blood velocity (FMBV), femoral mean artery diameter (FMAD), and femoral venous compliance (FVC; *P* > 0.05 in all cases). See Table 1 for mean values.

### 3.2. Training Adaptations

#### 3.2.1. Resting Blood Pressure

**Figure 1:**
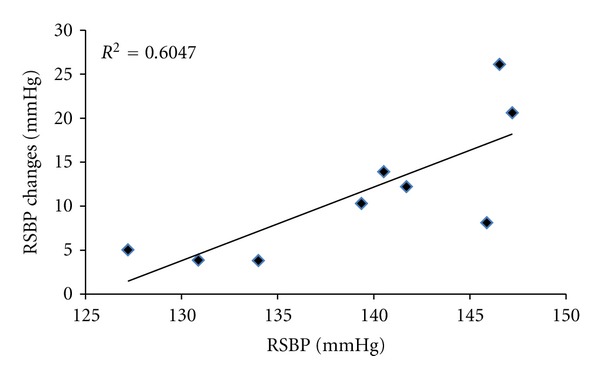
Regression line and plot of changes in training-induced resting systolic blood pressure (RSBP) and initial RSBP for the HI group following 8-week isometric exercise training.

**Figure 2:**
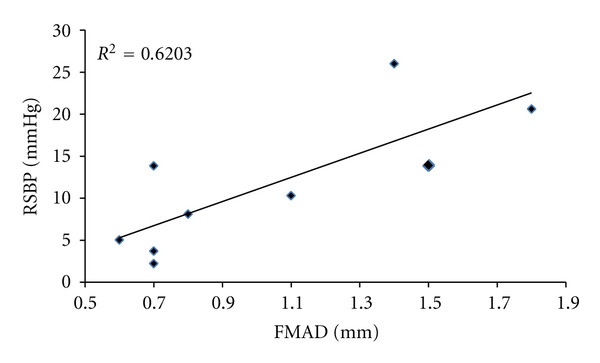
Regression line and plot of changes in femoral mean artery diameter (FMAD) and changes in resting systolic blood pressure (RSBP) for the HI group following 8 weeks of isometric exercise training.

There was a significant reduction in RMAP and RSBP after training in the HI group (from 98.3 ± 5.5 to 93.6 ± 6.6 mmHg for MAP; *P* < 0.01; from 138.7 ± 7.0 to 127.9 ± 8.0 mmHg, for SBP; *P* < 0.01; ANCOVA). However, there were no significant changes in RMAP or RSBP following the eight-week training intervention in either the control or the LO groups (all *P* > 0.05; ANCOVA). No significant changes in RDBP were evident in any group after training (all *P* > 0.05; ANCOVA). Individual analysis of all three groups using ANCOVA revealed no significant difference in the slopes (*P* > 0.05) for all blood pressure measures EMG relationships. There was also no significant difference in the elevation (intercept) of all blood pressure regression lines (*P* > 0.05) for the CON and LO groups. However, there was a significant difference in the elevation of the RSBP regression line (*P* < 0.01) for the HI group. See Table 2 for all mean resting blood pressure values. A significant correlation was identified between the initial RSBP values and the training-induced changes in RSBP (*r* = 0.79, *P* < 0.01; Figure 1). There was also a correlation evident (see Figure 2) between FMAD and RSBP changes (*r* = 0.79, *P* < 0.05) after training intervention. 

#### 3.2.2. Resting Heart Rate

A significant decrease in RHR was found in the HI group (from 71.0 ± 7.7 to 66.2 ± 5.4 b.min^−1^; *P* < 0.05). However, these reductions were not observed in the LO or the control groups (*P* > 0.05 in both cases). See Table 2 for all mean RHR values.

#### 3.2.3. Resting Femoral and Brachial Artery Measures

FABF increased significantly after training in the HI group (from 215.9 ± 39.2 to 298.0 ± 50.4 ml·min^−1^, *P* < 0.01). This appeared to have occurred via significant increases in FMBV from 7.2 ± 1.1 to 7.9 ± 1.0 cm·s^−1^ (*P* < 0.01) and FMAD from 8.1 ± 0.9 to 9.1 ± 1.1 mm (*P* < 0.01). These changes appeared to have occurred mostly between 4 and 8 weeks of training (See Table 3). There were no significant changes in these same variables in either the LO or the control groups (all *P* > 0.05). FVC also significantly increased compared to baseline values (from 2.19 ± 0.5 to 3.18 ± 0.5 U; *P *< 0.01). There were no significant changes in brachial artery measures (artery blood flow, mean blood velocity, and mean artery diameter) following training (all *P* > 0.05). See Table 3 for all resting artery values.

#### 3.2.4. Maximum Voluntary Contraction (MVC)

There was a significant increase (18.3 ± 18.1 Nm, *P* < 0.05) in MVC at the midtraining point (end of week 4) in the HI group. However, there were no further increases in MVC between the mid- and posttest (*P* > 0.05). Neither the LO (8.6 ± 32.6 Nm, *P* > 0.05) nor the control group (2.3 ± 5.1 Nm, *P* > 0.05) showed a significant change in their MVC at the end of the 8-week training period. 

## 4. Discussion

### 4.1. Summary of Results

The results of this study showed that training-induced reductions in resting blood pressure and concomitant vascular adaptations are dependent on isometric training intensity. The vascular adaptations comprised of increased resting femoral artery diameter, blood flow, blood velocity, and vascular conductance. These vascular changes only occurred in the higher-intensity training group and appeared to have occurred mostly between 4 and 8 weeks of training. These adaptations were not apparent in the vasculature of the untrained limbs (brachial artery) showing that the vascular adaptations were not only intensity-dependent, but were also localised and only became evident in the latter stages of the training intervention. 

### 4.2. The Role of Isometric Training Intensity

There are several factors associated with isometric training intensity that could be responsible for the intensity-dependent changes seen in the present study. 

The intensity of isometric exercise is known to influence the magnitude of central command, and therefore cardiovascular centre irradiation [1, 23]. Therefore, as intensity increases, there is a greater cardiac output, exercising systolic, and mean arterial blood pressure [23]. Because much of the effects of any type of exercise are known to be mediated via autonomic control, isometric exercise intensity, therefore, also influences the contribution of the sympathetic autonomic nervous system to the responses to such intense exercise [24]. Isometric exercise intensity is also known to affect the extent to which blood flow into the exercising muscle is occluded [25]. For example, although there have been conflicting reports, it appears that as isometric exercise intensity rises above 10–15% MVC, the degree to which blood flow is occluded increases in proportion to the force generated within the muscle [25, 26]. 

It has also been suggested that the intensity-dependent accumulation of exercising metabolites, although much lower than during dynamic exercise [25], plays an important role in metaboreceptor stimulation and thereby, could cause an intensity-dependent pressor response, during isometric exercise [27]. It is possible that the combination of powerful pressor response and high level of occlusion during higher-intensity isometric exercise is responsible for a marked effect on blood flow, and particularly on vascular shear rates, which might explain our localised vascular adaptations (this will be discussed in more detail, later in this section). It would follow that, at intensities where the pressor response is weak and the occlusion is incomplete, the stimulus for local vascular adaptations is too weak to see significant changes in resting blood pressure within 8 weeks. 

### 4.3. Systemic versus Local Intensity-Dependent Vascular Adaptations

The data reported in Figure 2 indicate a significant correlation between the decrease in RSBP and increases in FMAD. It is possible that these changes could have at least partly been caused by altered resting vascular tone, via modulation of sympathetic tone [2], especially since RHR was also reduced after training. Although both  *β*  and *α*-adrenergic receptors are involved in control of resting vascular tone, there have been recent studies that have demonstrated reduced vascular vasoconstrictor responsiveness to noradrenaline after training in animals [28]. This was explained as a training-induced attenuation of noradrenaline-mediated vasoconstriction, which is thought to occur through an upregulation of the endothelial *α*-receptor-nitric oxide synthase signalling pathway. However, similar findings have previously only been reported after resistance training in humans [29] and have been reported alongside *increases* in plasma concentrations of noradrenaline, not decreases, as shown in animals [28]. 

However, a more likely explanation is that at least part of the stimulus for such changes arises from changes in bioavailability and/or bioactivity of NO resulting from shear stresses during exercise [3, 5, 14]. As mentioned earlier, the augmented pressor response and the higher level of occlusion experienced during the higher-intensity training could have exposed the vasculature to especially high shear stresses, thereby, enhancing the release of NO. Not only would this stimulate vascular adaptations such as an increased lumen diameter [11, 15], but it also appears that the timecourse of these adaptations could explain some of our findings. Tinken et al. [13] suggested that there might be a biphasic pattern to the shear stress-induced adaptations in the local vasculature, comprising a NO-mediated increase in vascular function initially (2–4 weeks) and following longer-term training (8 weeks), the vasculature is remodelled to normalise shear stress [13, 14]. This would correspond with the vascular adaptations seen in our study which mostly occurred between 4 and 8 weeks of bilateral-leg isometric training. However, presently these explanations are somewhat speculative, not least because there is little previous evidence for such shear stress-induced adaptations in the femoral artery, which is thought to have reduced responsiveness to shear stress stimuli, compared to other vessels [30].

### 4.4. Autonomic Adaptations and Changes to Resting Heart Rate

To the authors' knowledge, no previous isometric training study has reported reduced resting heart rate following this type of training. The mechanisms responsible for the attenuated resting heart rate observed in the HI group are not easily explained. A combination of factors may be responsible, including changes to central command associated with reduced sympathetic nerve activity and a decrease in muscle mechano-/metabo-receptor afferent activity [31]. Indeed, participants in the HI group also demonstrated significant improvements in their MVC at the midpoint of training. Research suggests that trained muscles (possibly evident in the HI group at week 4) are more fatigue-resistant and muscle fibres may be recruited more synchronously [32]. Therefore, participants may experience reduced accumulation of metabolites [27, 32], thereby reducing the afferent metabo-receptor response [27, 33]. 

However, whilst this could explain a reduced exercising heart rate, it does not explain the reduced heart rate at rest. Some previous researchers have reported changes in heart rate variability after isometric training [2], a marker for autonomic adaptations, which may better explain the heart rate data. Heart rate variability (and blood pressure variability) analyses showed increased vagal activity in that study. Other researchers have reported no change in mean sympathetic nerve activity [34] after such training (although their reductions in resting blood pressure were modest). However, more-recent work by Millar et al. [35] has shown improved neurocardiac regulation immediately after a single bout of isometric handgrip exercise. 

### 4.5. The Influence of the Initial Resting Blood Pressure Being 138 mmHg

It is possible that the differences in the magnitude of the reduction in resting blood pressure may be due, in part, to the initial resting levels. Indeed the data presented in Figure 1 identified a significant correlation between these measures. The majority of training studies that have noted reductions in resting blood pressure following isometric exercise training in older individuals have used participants who were either hypertensive [5, 6] or high normotensive [2, 36]. Interestingly, the majority of the observed blood pressure changes in these and the present training study tend to be greater than those seen when using normotensive adults. Results of training studies recruiting predominantly younger normotensive subjects report reductions in systolic blood pressure of approximately 4-5 mmHg [7, 34]. The only other study to investigate the blood pressure responses to isometric bilateral-leg extension over an eight-week period reported systolic blood pressure reductions of 5 mmHg [4], approximately half of that observed in this investigation. 

As hypertension is associated with endothelial dysfunction, it is possible that the mechanisms responsible for the observed reductions in resting blood pressure in participants with elevated blood pressure may differ to those seen in normotensive adults. Previous research using hypertensive adults [5, 36] have reported elevations in endothelial-dependent vasodilation of the trained limb following isometric exercise training. They noted that shear stress mediated increased bioactivity and/or bioavailability of NO could be responsible for the training induced improvements in endothelial function. Furthermore, as these studies reported no changes in endothelial-independent vasodilation, it was suggested that these changes were influenced by functional rather than structural adaptations [5]. However, as the elevations in endothelial-dependent vasodilation were only evident in the trained limb rather than systemically, and blood pressure is primarily mediated by resistance vessels, it is unlikely that the changes in femoral structure would be fully responsible for the improvements in after training resting blood pressure in hypertensive individuals. Although, it is possible that the elevations in the NO-dilator system may be involved in the systemic reduction in resting blood pressure [36]. 

### 4.6. The Influence of Age on the Adaptations in the Vasculature to Isometric Training

To date, isometric training studies have either used young healthy adults ≤40 yrs [1, 4, 34] or older adults ≥60 yrs [2, 5, 37], when investigating blood pressure adaptation and its potential causes, with no research specifically focusing on middle-aged adults (45–60 yrs). Ageing is suggested to be associated with increased physiological stress during submaximal exercise, a reduced capacity to perform exercise [38] and elevated levels of resting heart rate and blood pressure [39]. These changes have been explained by age-related functional decline of bodily systems including the cardiovascular and musculoskeletal systems [40]. More specifically, research has reported a loss of large artery compliance and elevated sympathetic vasoconstriction [41, 42], between the ages of 30–60 [38], with a more pronounced functional decline thereafter [43]. All of these physiological changes could theoretically influence the responses and adaptations of individuals in this age group to isometric training.

## 5. Conclusion

The findings of the present study showed that training-induced reductions in resting blood pressure were associated with changes in the local vasculature, which were dependent on isometric training intensity. These changes occurred mostly in the later stages of training (between 4 and 8 weeks) and were not evident in untrained limbs. 

## Figures and Tables

**Table 1:** Control and trained groups resting baseline data.

	CON	LO	HI
Age (yrs)	53.4 ± 5.0	53.6 ± 5.5	54.6 ± 5.5
Height (cm)	181.2 ± 4.4	182.8 ± 6.3	180.2 ± 5.1
Body mass (Kg)	92.1 ± 2.2	88.7 ± 2.9	89.6 ± 3.3
MVC (Nm)	304.6 ± 88.1	289.9 ± 49.9	301.2 ± 87.2
RHR (bpm)	69.0 ± 5.8	69.1 ± 5.3	71.0 ± 7.7
RSBP (mmHg)	139.1 ± 2.2	137.3 ± 5.3	138.7 ± 7.0
RDBP (mmHg)	78.9 ± 10.3	78.3 ± 5.5	78.2 ± 5.5
RMAP (mmHg)	98.9 ± 7.1	97.9 ± 5.2	98.3 ± 5.5
FABF (mL/min)	214.7 ± 49.2	219.7 ± 50.7	216.9 ± 39.2
FMBV (cm/s)	7.1 ± 1.3	7.2 ± 1.9	7.2 ± 1.1
FMAD (mm)	8.1 ± 0.9	7.9 ± 0.5	8.1 ± 0.9
FVC (U)	2.27 ± 0.3	2.25 ± 0.6	2.19 ± 0.5

Values are means ± SD (CON group *n* = 10; LO group *n* = 10; HI group *n* = 10). MVC: maximum voluntary contraction; RHR: resting heart rate; RSBP: resting systolic blood pressure; RDBP: resting diastolic blood pressure; RMAP: resting mean arterial pressure; FABF: femoral artery blood flow; FMBV: femoral mean blood velocity; FMAD: femoral mean arterial diameter; FCV: femoral vascular conductance.

**Table 2:** Group mean changes in resting blood pressure and heart rate measures over the 8-week training period.

Group		Pre-training mean ± S.D	Midtraining mean ± S.D	Post-training mean ± S.D
HI	RSBP (mmHg)	138.7 ± 7.0	134.6 ± 8.4	127.9 ± 8.0^†^
	RDBP (mmHg)	78.2 ± 5.5	77.3 ± 5.6	76.6 ± 7.4
	RMAP (mmHg)	98.3 ± 5.5	96.4 ± 5.5	93.6 ± 6.6^†^
	RHR (b·m^−1^)	71.0 ± 7.7	67.9 ± 5.1	66.2 ± 5.4*

LO	RSBP (mmHg)	137.3 ± 5.3	137.8 ± 7.9	136.5 ± 5.9
	RDBP (mmHg)	78.3 ± 5.5	80.3 ± 7.6	79.4 ± 5.6
	RMAP (mmHg)	97.9 ± 5.2	99.5 ± 6.9	98.4 ± 5.4
	RHR (b·m^−1^)	69.1 ± 5.3	68.6 ± 3.4	68.8 ± 5.4

CON	RSBP (mmHg)	139.1 ± 2.2	139.5 ± 5.4	138.9 ± 4.4
	RDBP (mmHg)	78.9 ± 10.3	79.9 ± 6.2	78.8 ± 7.4
	RMAP (mmHg)	98.9 ± 7.1	99.8 ± 4.6	98.8 ± 5.0
	RHR (b·m^−1^)	69.0 ± 5.8	69.1 ± 5.7	68.2 ± 5.4

Values are means ± SD (CON group *n* = 10; LO group *n* = 10; HI group *n* = 10). RSBP: resting systolic blood pressure; RDBP: resting diastolic blood pressure; RMAP: mean arterial pressure; RHR: resting heart rate.

*P value < 0.05; ^†^ *P* value <0.01.

**Table 3:** Group mean changes in resting femoral and brachial artery measures over the 8-week-training period.

Group	Artery Measures	Pre-training mean ± S.D	Midtraining mean ± S.D	Post-training mean ± S.D
	FABF (mL/min)	215.9 ± 39.2	219.2 ± 36.6	298.0 ± 50.4^†^
HI	FMBV (cm/s)	7.2 ± 1.1	7.3 ± 1.3	7.9 ± 1.0^†^
	FMAD (mm)	8.1 ± 0.9	8.2 ± 1.1	9.1 ± 1.1^†^

	FABF (mL/min)	219.8 ± 50.7	219.2 ± 50.7	224.8 ± 60.7
LO	FMBV (cm/s)	7.2 ± 1.9	7.0 ± 1.9	7.1 ± 1.9
	FMAD (mm)	7.9 ± 0.5	7.9 ± 0.5	7.9 ± 0.4

	FABF (mL/min)	214.7 ± 49.2	219.1 ± 52.0	216.2 ± 48.6
CON	FMBV (cm/s)	7.1 ± 1.3	7.2 ± 1.3	7.0 ± 1.2
	FMAD (mm)	8.1 ± 0.9	8.1 ± 1.0	8.2 ± 0.9

	BABF (mL/min)	50.7 ± 19.7	51.6 ± 19.7	53.3 ± 21.1
HI	BMBV (cm/s)	5.9 ± 1.2	5.8 ± 1.3	5.8 ± 1.3
	BMAD (mm)	4.3 ± 0.7	4.4 ± 0.8	4.4 ± 0.7

	BABF (mL/min)	49.8 ± 11.9	49.6 ± 13.2	49.6 ± 12.8
LO	BMBV (cm/s)	5.8 ± 1.2	5.9 ± 1.6	5.9 ± 1.3
	BMAD (mm)	4.3 ± 0.4	4.2 ± 0.4	4.3 ± 0.4

	BABF (mL/min)	49.0 ± 23.1	50.4 ± 23.9	49.8 ± 24.0
CON	BMBV (cm/s)	6.1 ± 1.4	6.1 ± 1.8	5.9 ± 1.2
	BMAD (mm)	4.1 ± 0.7	4.1 ± 0.6	4.1 ± 0.6

Values are means ± SD (CON group *n* = 10; LO group *n* = 10; HI group *n* = 10). FABF: femoral artery blood flow; FMBV: femoral mean blood velocity; FMAD: femoral mean arterial diameter; BABF: brachial artery blood flow; BMBV: brachial mean blood velocity; BMAD: brachial mean arterial diameter.

*P value < 0.05; ^†^ *P* value < 0.01.
